# Potential interaction between the oral microbiota and COVID-19: a meta-analysis and bioinformatics prediction

**DOI:** 10.3389/fcimb.2023.1193340

**Published:** 2023-06-07

**Authors:** Li Tan, Meng-Mei Zhong, Qiong Liu, Yun Chen, Ya-Qiong Zhao, Jie Zhao, Marie Aimee Dusenge, Yao Feng, Qin Ye, Jing Hu, Ze-Yue Ou-Yang, Ying-Hui Zhou, Yue Guo, Yun-Zhi Feng

**Affiliations:** ^1^ Department of Stomatology, The Second Xiangya Hospital, Central South University, Changsha, Hunan, China; ^2^ National Clinical Research Center for Metabolic Diseases, Hunan Provincial Key Laboratory of Metabolic Bone Diseases, and Department of Metabolism and Endocrinology, The Second Xiangya Hospital of Central South University, Changsha, Hunan, China

**Keywords:** oral microbiota, COVID-19, meta-analysis, bioinformatics prediction, SARS-CoV-2

## Abstract

**Objectives:**

The purpose of this study was to evaluate available evidence on the association between the human oral microbiota and coronavirus disease 2019 (COVID-19) and summarize relevant data obtained during the pandemic.

**Methods:**

We searched EMBASE, PubMed, and the Cochrane Library for human studies published up to October 2022. The main outcomes of the study were the differences in the diversity (α and β) and composition of the oral microbiota at the phylum and genus levels between patients with laboratory-confirmed SARS-CoV-2 infection (CPs) and healthy controls (HCs). We used the Human Protein Atlas (HPA), Gene Expression Profiling Interactive Analysis (GEPIA) database, Protein−protein interaction (PPI) network (STRING) and Gene enrichment analysis (Metascape) to evaluate the expression of dipeptidyl peptidase 4 (DPP4) (which is the cell receptor of SARS CoV-2) in oral tissues and evaluate its correlation with viral genes or changes in the oral microbiota.

**Results:**

Out of 706 studies, a meta-analysis of 9 studies revealed a significantly lower alpha diversity (Shannon index) in CPs than in HCs (standardized mean difference (SMD): -0.53, 95% confidence intervals (95% CI): -0.97 to -0.09). Subgroup meta-analysis revealed a significantly lower alpha diversity (Shannon index) in older than younger individuals (SMD: -0.54, 95% CI: -0.86 to -0.23/SMD: -0.52, 95% CI: -1.18 to 0.14). At the genus level, the most significant changes were in Streptococcus and Neisseria, which had abundances that were significantly higher and lower in CPs than in HCs based on data obtained from six out of eleven and five out of eleven studies, respectively. DPP4 mRNA expression in the oral salivary gland was significantly lower in elderly individuals than in young individuals. Spearman correlation analysis showed that DPP4 expression was negatively correlated with the expression of viral genes. Gene enrichment analysis showed that DPP4-associated proteins were mainly enriched in biological processes, such as regulation of receptor-mediated endocytosis of viruses by host cells and bacterial invasion of epithelial cells.

**Conclusion:**

The oral microbial composition in COVID-19 patients was significantly different from that in healthy individuals, especially among elderly individuals. DPP4 may be related to viral infection and dysbiosis of the oral microbiome in elderly individuals.

## Introduction

Coronavirus disease 2019 (COVID-19), caused by a respiratory virus known as SARS-COV-2, became the causative pathogen of a global epidemic in late 2019 (Amano, 2007; Zhu et al., 2020). The most frequent causes of death associated with COVID-19 are respiratory failure, pneumonia, septic shock, acute respiratory distress syndrome and multiple organ failure (Zhou et al., 2020). Immune dysregulation and bacterial coinfection may lead to the aforementioned conditions and are known to contribute to the high mortality rate of COVID-19 (Chen et al., 2020; Cox et al., 2020; Mirzaei et al., 2020; O'Driscoll et al., 2021; Wang and Perlman, 2022). However, the role of oral bacteria in immune dysregulation and bacterial coinfection in COVID-19 is pertinent yet overlooked (Patel and Sampson, 2020; Haran et al., 2021; Ghosh et al., 2022).

On the one hand, previous studies revealed that the oral microbiome plays a role in regulating innate and adaptive immunity (Zhao et al., 2021; Gao et al., 2022). Furthermore, recent studies have shown that lipopolysaccharides (LPS)-producing bacteria in the oral cavity of patients with COVID-19 can have an inflammatory effect on the host immune system (Bonnington and Kuehn, 2014; Larsen, 2017; Ren et al., 2021). For instance, some studies have revealed that the oral microbiota of COVID-19 patients has a high level of Prevotella and Veillonella, which may stimulate the expression of inflammatory factors such as IL-6, IL-23, and IL-1 (Segal et al., 2013; van den Bogert et al., 2014; Segal et al., 2016; Khan and Khan, 2020; Iebba et al., 2021). Moreover, recent studies have shown that the clinical symptoms of COVID-19, such as loss of taste, difficulty breathing, and sore throat, are not caused by direct viral damage but may be related to chronic inflammation and immune-subversion induced by dysbiosis of the oral microbiota (Gupta and Gupta, 2021; Ho et al., 2022; Rafiqul Islam et al., 2022).

On the other hand, the oral microbiome has been shown to be closely associated with bacterial coinfection in COVID-19 patients (Bao et al., 2020). A recent study showed that a variety of oral opportunistic pathogens have been detected in the bronchoalveolar lavage fluid of patients with COVID-19 (Shen et al., 2020). Microbes associated with the oral microbiome may increase the possibility of bacterial coinfection in COVID-19 patients because aspiration of the oral cavity and lungs is an important cause of many infectious diseases (Mammen et al., 2020). Moreover, pulmonary hypoxia is a typical symptom of COVID-19, which provides a very favourable anaerobic condition for coinfection with oral bacteria, since most oral microbes are facultative anaerobic bacteria or anaerobes (Bao et al., 2020). Some studies have also shown that dysbiosis of the oral microbiome in addition to considerable composition changes can enrich for opportunistic pathogens and increase the risk of bacterial coinfection in COVID-19 patients (Prasad et al., 2022).

Based on the above findings, the oral microbiome is closely related to immune dysregulation and bacterial coinfections in COVID-19. Therefore, obtaining data on the oral microbiome and elucidating the composition of the oral microbiota associated with COVID-19 disease may provide ideas for identifying the potential pathogenic bacteria that may aggravate immune dysregulation and bacterial coinfection in COVID-19 patients and enable the investigation of new ideas for preventing and reversing its progression to reduce the mortality of COVID-19.

To determine the potential role of the oral microbiome in COVID-19, a number of studies have investigated the features of oral microbes in COVID-19 patients. However, outcomes such as the composition (at the phylum and genus levels) and diversity (α and β) of the oral microbiome often inconsistent to some extent (Iebba et al., 2021; Miller et al., 2021; Ren et al., 2021; Wu et al., 2021). Until now, the features of oral microbes in COVID-19 patients had not been analysed by a meta-analysis. Therefore, it is meaningful and necessary to conduct a summative and evidence-based meta-analysis of the recent study results in this area.

Moreover, recent studies have shown that some primary receptors for SARS-CoV-2, such as dipeptidyl peptidase 4 (DPP4) and angiotensin converting enzyme 2 (ACE2), are highly expressed in intestinal epithelial cells and may modify the gut microbiome and increase the levels of opportunistic pathogens in COVID-19 patients, which further leads to immune dysregulation and increases the mortality rate of COVID-19 (Olivares et al., 2018; Liskova et al., 2021; Penninger et al., 2021; Posadas-Sánchez et al., 2021). However, it is still unclear whether their expression levels in oral tissues are related to changes in the oral microbiome in COVID-19 patients. Therefore, we also conducted bioinformatics analysis to predict this correlation.

## Methods

The protocol used in this study has been registered in the International Platform of Registered Systematic Review and Meta-analysis Protocols (INPLASY) platform (INPLASY2022100113), and this article followed the guidelines of the Preferred Reporting Items for Systematic Reviews and Meta-Analyses (PRISMA) 2020 statement (Page et al., 2021).

### Eligibility criteria

Inclusion criteria were studies (case−control studies, cross-sectional studies, cohort studies, and clinical trials) that compared the composition of the oral microbiome using high-throughput analyses (e.g., 16S rDNA/rRNA sequencing) between laboratory-confirmed SARS-CoV-2 infection patients (CPs) and healthy controls (HCs) (age ≥ 18 years). Exclusion criteria were reviews, commentaries, short surveys, case reports, and letters. An additional exclusion criterion was focus on specific diseases.

### Information sources

Three well-known databases (PubMed, EMBASE, Cochrane Library) related to previously published studies on COVID-19 and oral microbiomes were screened. Furthermore, these databases were searched for relevant articles without limits of time-frame or language (last updated October 2022) to ensure that the obtained data were complete. Google translate was used to translate any non-English publications.

### Literature search

The search strategy and focus involved the use of the following key words: ‘COVID-19’, ‘SARS-CoV-2’, ‘Coronavirus Disease 19’, ‘Coronavirus Disease 19’, ‘oral microbes’, ‘oral microbiota’ and ‘oral bacteria’, and the list of studies was expanded using the author’s knowledge or references from the obtained studies. Subsequently, Boolean and truncation operations (‘OR’, ‘AND’) were employed to implement search strategies based on sensitivity and specificity and were adapted for each database. For example, the PubMed search strategy is shown in **Table 1** (we have provided the detailed search strategy used for EMBASE and Cochrane databases in **Tables S1**, **2**).

**Table 1:** PubMed search strategy.

PubMed	Search Strategy (October, 2022)	Items
#1	((((((((((COVID-19[MeSH Terms]) OR (SARS-CoV-2[MeSH Terms])) OR (COVID-19[Title/Abstract])) OR (severe acute respiratory syndrome coronavirus 2[Title/Abstract])) OR (SARS-CoV-2[Title/Abstract])) OR (SARS2[Title/Abstract])) OR (wuhan coronavirus[Title/Abstract])) OR (coronavirus[Title/Abstract])) OR (novel coronavirus[Title/Abstract])) OR (nCoV[Title/Abstract])) OR (coronavirus disease 2019[Title/Abstract])	314952
#2	((((microbiota[MeSH Terms]) OR (oral microbiome[Title/Abstract])) OR (oral flora[Title/Abstract])) OR (oral bacteria[Title/Abstract])) OR (oral microbiota[Title/Abstract])	72040
#3	#1 AND #2	572

### Study selection

After removing duplicate articles, two reviewers (QL & YC) evaluated the titles/abstracts and full text independently by using the above criteria to select appropriate studies. In case of any dispute between the two reviewers, a third reviewer (YQ-Z) participated in the discussion and resolved the disagreements. For quality evaluation and evidence synthesis, the data obtained from the selected studies were extracted by the same reviewer using a standardized prepiloted form.

### Features of the data

**Figure 1:**
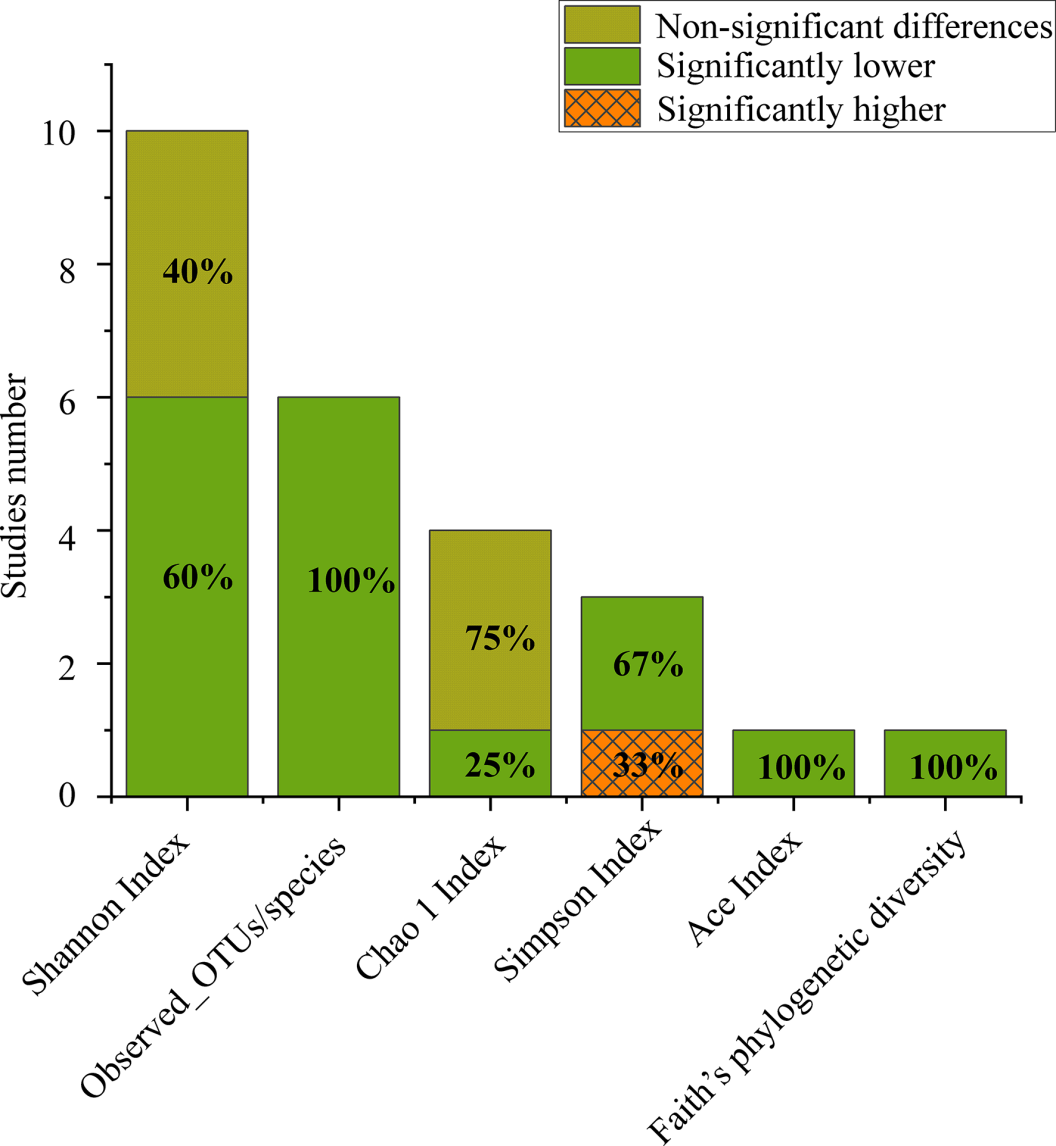
Number of studies that reported alpha diversity, as significantly higher (orange grid), significantly lower (grey) or nonsignificant differences (turquoise) when comparing CPs to HCs.

**Figure 2:**
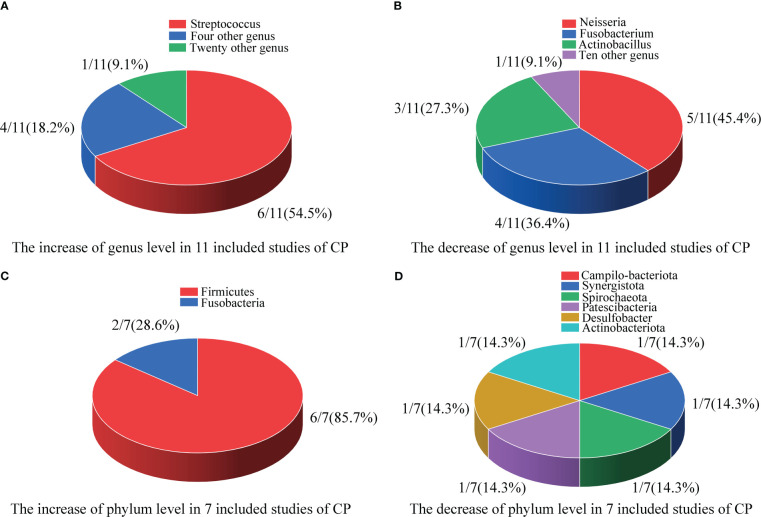
Differences in the composition of the oral microbiome at the genus and phylum levels. **(A)** The increase at the genus level in 11 included studies; **(B)** The decrease at the genus level in 11 included studies; **(C)** The increase at the phylum level in 7 included studies; **(D)** The decrease at the phylum level in 7 included studies.

Data extraction was performed by two reviewers using spreadsheets (Excel 2007, Microsoft^©^, CA, USA). Divergent views were discussed until consensus was reached. The data collected included first author name, year of publication, country where the study was performed, type of study, study population, average age, sex, COVID-19 severity, comorbidity, microbiome analysis techniques, type of sample, whether antibiotics were used, the diversity (α and β) of the oral microbiota and the composition of the oral microbiota at different phyla and genus levels in the context of COVID-19. All of the above information is summarized in **Tables 2**–**5**; **Table S3**; **Figures 1**, **2**.

**Table 2:** Age and gender of included studies.

	Details of age and gender						
Study	Age of CP	Age of HC			Gender of CP	Gender of HC	
Wu et al., 2021	48.5 (32.0-64.0)	41.5 (36.3-51.0)			30M/22F	31M/13F	
Soffritti et al., 2021	71.1 ± 18.4	66.5 ± 18.8			20M/19F	22M/14F	
Shi et al., 2022	Unknown	Unknown			10M/10F	10M/10F	
Schult et al., 2022	62 ± 15	63 ± 12			59M/49F	15M/11F	
Ren et al., 2021	48.40 ± 13.90	44.88 ± 11.35			20M/28F	37M/63F	
Miller et al., 2021	56.5 ± 16.1	56.2 ± 16.8			24M/29F	36M/23F	
Rafiqul Islam et al., 2022	> 18	> 18			Unknown	Unknown	
Iebba et al., 2021	66 ± 15	66 ± 15			20M/6F	36M/23F	
Gupta et al., 2022	47/48.5	45/43.5			20M/10F	14M/10F	
Cui et al., 2022	48.93 ± 16.21	44.15 ± 11.91			10M/28F	55M/95F	
Callahan et al., 2022	47.2 ± 13.3	52.3 ± 15.7			7M/9F	36M/54F

M represents males; F represents females.

**Table 3:** Characteristics of the included studies.

Studies	Year	Country		Type of study	Study population	Oral microbiota analysis technique	Samples	
Wu et al., 2021	2021	China		Case-control	CP (140), HC (44)	16S rRNA gene sequencing	Throat swab samples	
Soffritti et al., 2021	2021	Italy		Cross-sectional	CP (39), HC (36)	Whole-genome sequencing	Oral rinse samples	
Shi et al., 2022	2022	China		Case-control	CP (10), HC (10)	16S rRNA gene sequencing	Throat swabs	
Schult et al., 2022	2022	Germany		Case-control	CP (108), HC (26)	16S rRNA gene sequencing	Saliva samples	
Ren et al., 2021	2021	China		Case-control	CP (48), HC (100)	16S rRNA gene sequencing	Tongue coating samples	
Miller et al., 2021	2021	USA		Case-control	CP (53), HC (59)	16S rRNA gene sequencing	Saliva samples	
Rafiqul Islam et al., 2022	2022	Bangladesh		Cross-sectional	CP (22), HC (19)	Whole-genome sequencing	Saliva samples	
Iebba et al., 2021	2021	Italy		Case-control	CP (26), HC (15)	16S rRNA gene sequencing	Tongue coating samples	
Gupta et al., 2022	2022	India		Case-control	CP (30), HC (24)	16S rRNA gene sequencing	Saliva samples	
Cui et al., 2022	2022	China		Cohort	CP (38), HC (150)	16S rRNA gene sequencing	Tongue coating samples	
Callahan et al., 2022	2022	USA		Case-control	CP (16), HC (90)	16S rRNA gene sequencing	Saliva samples	

**Table 4:** Differences in the relative abundance of bacteria at genus level.

Studies	Main alternations of CP
Wu et al., 2021	Veillonella↑, Campylobacter↑, Granulicatella↑, Kingella↑, Filifactor↑,Neisseria↓, Corynebacterium↓, Actinobacillus↓, Moryella↓, Aggregatibacter↓.
Soffritti et al., 2021	Enterococcus↑, Enterobacter↑,Streptococcus↑, Veillonella↑, Prevotella↑, Lactobacillus↑,Capnocytophaga↑, Porphyromonas↑, Abiotrophia↑, Aggregatibacter↑, Atopobium↑,Rothia↓, Haemophilus↓, Parvimonas↓, Fusobacterium↓, Gemella↓.
Shi et al., 2022	Campylobacter↑,Streptococcus↑, Haemophilus↑, Gemella↑, Aggregatibacter↑, Prevotella↓, Veillonella↓, Actinobacillus↓.
Schult et al., 2022	Parabacteroides↑, Lachnoclostridium↑, Blautia↓, Faecalibacterium↓, Ruminococcus↓.
Ren et al., 2021	Leptotrichia↑, Selenomonas↑, Granulicatella↑, megasphaera↑, Haemophilus↓, Porphyromonas↓, Fusobacterium↓.
Miller et al., 2021	Streptococcus↑, Actinomyces↑,Treponema↑, Prevotella↓.
Rafiqul Islam et al., 2022	Streptococcus↑, Rothia↑, Klebsiella↑,Veillonella↑, Enterococcus↑, Neisseria↓, Prevotella↓,Haemophilus↓, Porphyromonas↓.
Iebba et al., 2021	Neisseria↓.
Gupta et al., 2022	Streptococcus↑, Veillonella↑, Prevotella↑, Bacillus↑, Klebsiella↑, Idiomarina↑, Acinetobacter↑, Arenibacter↑, Gemella↑, Chryseobacterium↑, Capnocytophaga↑,Neisseria↓,Haemophilus↓, Pseudomonas↓, Lautropia↓, Rothia↓, Leptotrichia↓, Porphyromonas↓, Actinobacillus↓, Granulicatella↓, Fusobacterium↓, Aggregatibacter↓, Alloprevotella↓, Selenomonas↓.
Cui et al., 2022	Prevotella↑, Streptococcus↑, Veillonella↑, Neisseria↓, Porphyromonas↓, Fusobacterium↓, Haemophilus↓, Rothia↓.
Callahan et al., 2022	Prevotella↑, Bergeyell↑, Schaalia↑, Bacteroidete↑, Neisseria↓.

The symbols "↑" and "↓" respectively represent the increase and decrease in the relative abundance of bacteria at the genus level.

**Table 5:** Differences in the relative abundance of bacteria at phylum level.

Studies	Main alternations of CP
Soffritti et al., 2021	Bacteroidetes↑, Firmicutes↑, Proteobacteria↓, Actinobacteria↓.
Shi et al., 2022	Firmicutes↑, Fusobacteria↑, Actinobacteria↑,Proteobacteria↓.
Schult et al., 2022	Firmicutes↑,Proteobacteria↑.
Ren et al., 2021	Firmicutes↑, Fusobacteria↑, Bacteroidetes↓, Proteobacteria↓.
Rafiqul Islam et al., 2022	Firmicutes↑, Proteobacteria↓.
Gupta et al., 2022	Firmicutes↑,Proteobacteria↓,Fusobacteriota↓,Campilo-bacteriota↓, Synergistota↓, Spirochaeota↓, Patescibacteria↓, Desulfobacter↓.
Cui et al., 2022	Fusobacteriota↑, Bacteroidetes↓, Actinobacteriota↓.

Four studies have not mentioned the information about differences in the relative abundance of bacteria at phylum level. (Iebba et al., 2021; Miller et al., 2021; Wu et al., 2021; Callahan et al., 2022),

The symbols "↑" and "↓" respectively represent the increase and decrease in the relative abundance of bacteria at the phylum level.

### Data synthesis

All included studies were synthesized narratively according to the inclusion criteria, but meta-analyses were limited to quantifying results as the means and standard differences or enabling manual calculations using Excel 2010 (Washington, Microsoft, USA). For studies in which the outcome data were presented as the median, minimum and maximum values and the first and third quartiles, the method described by McGrath et al. (2020) was used to convert these data from the reported summary data into the mean or standard deviation for analysis. If none of the above methods could be used to obtain the raw data to be analysed, we sent an email to ask the author to provide these data. ImageJ 1.38e software (Wayne Rasband, National Institutes of Health, USA) was used to obtain raw data that were presented in graphs but not provided by the author. To further study the influence of age and the use of antibiotics on the oral microbiota, a subgroup meta-analysis was performed to compare CPs and HCs. Outcomes are shown in forest plots, where the edges and middle of the rhombus represent the 95% confidence interval (95% CI) and the standard mean difference (SMD) point estimate, respectively. The 95% CI and point estimate for each study are presented as a horizontal line and a central symbol, respectively. Chi-squared analyses and I^2^ scores were calculated to analyse homogeneity. Random-effects models were used for the meta-analysis. All calculations were carried out using Review Manager 5.4.

### Risk of bias assessment

Two independent investigators (LT and QY) used a Cochrane-based (Cochrane) Modified Bias in Trials of Nonrandomized Interventions (ROBINS-I) tool to assess bias (Sterne et al., 2016). Then, the investigators discussed and negotiated with a third author as appropriate to resolve disagreements. The revision of the ROBINS-I tool includes the following six domains of biases (1): confounding (2), participant selection (3), exposure assessment (4), missing data (5), outcome measures, and (6) selective reporting of the results, in addition to indicating issues that can facilitate the judgement of potential risk of bias for each domain. The overall risk of bias was assessed as low, moderate or serious. If at least one domain was identified as having a serious risk and the other was not considered to have serious risk, the overall risk was considered serious. If all regions were considered to be at low risk, the overall rating was low. If all areas were of low or moderate risk, the overall rating was moderate.

### Publication bias

Since only 9 articles were selected for meta-analysis, it was not reasonable to use funnel plots and related statistical tests for analysis (as tests for publication bias only have sufficient power when there are at least 10 studies).

### Sensitivity analysis

Sensitivity analysis was performed by omitting each study from the meta-analysis until heterogeneity decreased significantly. If there was no difference in the meta-analysis synthesis results before and after the exclusion of the relevant literature, the original synthesis results were considered to be relatively stable.

### Analysis of ACE2 and DPP4 mRNA and protein expression data in oral tissues

The distribution and clinical characteristics of ACE2 and DPP4 expression in healthy individuals were derived from genotype tissue expression (GTEx), which includes mRNA expression data obtained from donors after death. GTEx data were downloaded from the Human Protein Atlas (HPA, https://www.proteinatlas.org/) and analysed and visualized using the log2(n+1) scale. Normal oral tissues were evaluated by immunohistochemistry. Immunohistochemical images and antibody data were also obtained from HPA. The mean expression levels of ACE2 and DPP4 mRNA and differences across elderly (> 60 years old) and young (< 60 years old) groups according to the World Health Organization’s definition of elderly individualswere compared using Student’s t test, and all results (two-sided) were considered significant when *P <*0.05 (Fritz et al., 2017).

### Gene correlation analysis

Web-based Gene Expression Profiling Interactive Analysis (GEPIA, http://gepia.cancer-pku.cn/) was used to conduct gene correlation analysis. GEPIA was used to conduct a paired gene association study using the Cancer Genome Atlas (TCGA) and the GTEx database. In this study, only the GTEx gene was evaluated. The correlation between the levels of DPP4 and viral genes was analysed by Spearman’s correlation.

### Protein−protein interaction network and gene enrichment analysis

The Search Tool for the Retrieval of Interacting Genes/Proteins (STRING, https://string-db.org/) is a website that is used to predict the interaction partners of input proteins according to a combined score (Szklarczyk et al., 2019). The 10 proteins that had the highest combined score with DPP4 in STRING were used to establish a PPI network. Then, a gene enrichment analysis using Metascape (https://metascape.org/) was conducted to predict the biological processes in which DPP4 and its 10 predicted partners were involved (Zhou et al., 2019).

## Results

### Selected studies

**Figure 3:**
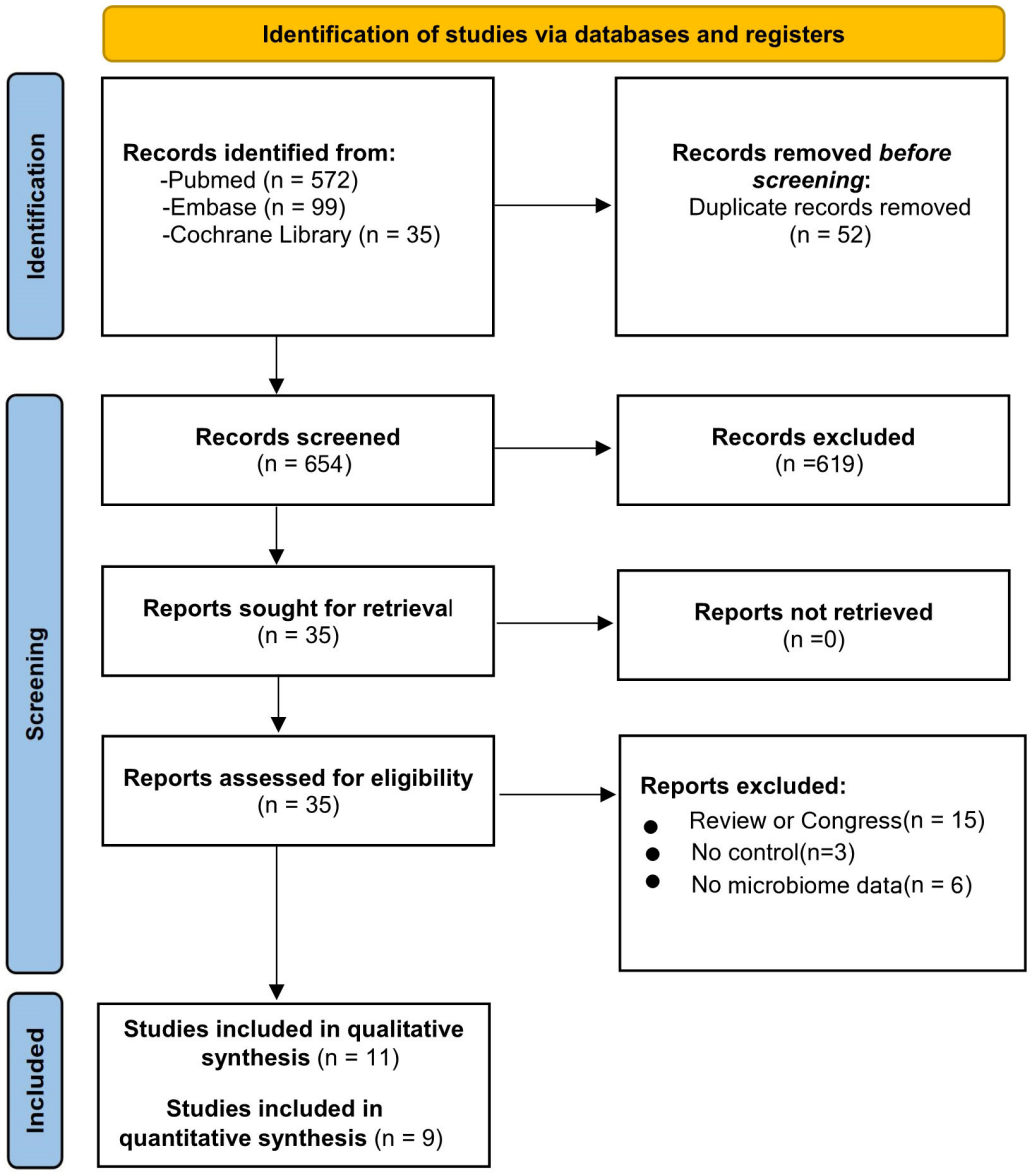
Flow diagram based on PRISMA 2020 guidelines.

Following the initial search, 706 studies were selected in total. Of those, 52 were eliminated because of duplications, and 654 of the remaining studies were evaluated according to the inclusion criteria (**Figure 3**). Following the full-text screening, 11 studies met the inclusion criteria (Iebba et al., 2021; Miller et al., 2021; Ren et al., 2021; Soffritti et al., 2021; Wu et al., 2021; Callahan et al., 2022; Cui et al., 2022; Gupta et al., 2022; Rafiqul Islam et al., 2022; Schult et al., 2022; Shi et al., 2022). Only 9 of these studies provided a meta-analysis with enough quantitative data (Miller et al., 2021; Ren et al., 2021; Soffritti et al., 2021; Callahan et al., 2022; Cui et al., 2022; Gupta et al., 2022; Rafiqul Islam et al., 2022; Schult et al., 2022; Shi et al., 2022). **Figure 3** also provides a detailed explanation of why the 24 publications were rejected and excluded from the full-text review.

### Study characteristics

A total of 442 CPs and 573 HCs were examined in the 11 included studies. The age and sex of the individuals in each study are shown in **Table 2**. Most of the studies were case–control (n = 8), followed by cross-sectional (n = 2) and cohort (n = 1) studies. Four of the studies were executed in China; 2 in the United States (US) and Italy; and 1 in Bangladesh, Germany, and India (**Table 3**).

Ten articles adopted the method of 16S rRNA gene sequencing followed by whole-genome sequencing (n = 1) for oral microbiome analysis, and all of them were published between 2021 and 2022. The most common samples used for sequencing the oral microbiome were saliva samples (n = 5), followed by tongue coating samples (n = 3) and then throat swab samples (n = 2) or oral rinse samples (n=1) (**Table 3**).

Three studies showed that the comorbidity that was common among the CPs was hypertension, while the other eight studies did not mention comorbidities (**Table S3**). Five studies showed that different numbers of CPs took antibiotics during the examination of the oral microbiota, five studies showed that none of the CPs took antibiotics, and one study did not mention whether the CPs took antibiotics (**Table S3**). Five studies graded the severity of COVID-19 in CPs through multiple evaluation methods, the other five did not mention the strategy used to grade severity, and one study included CPs who had just recovered (**Table S3**).

#### Alpha diversity analysis

**Figure 4:**
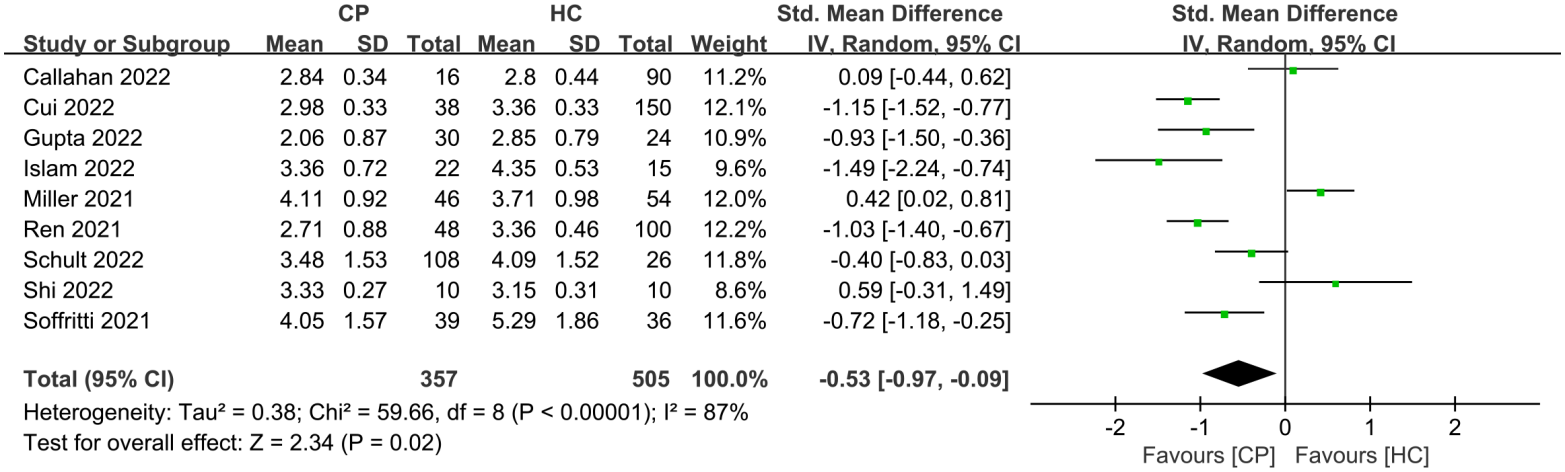
Forest plots for the Shannon index comparing the oral microbiome of CPs with that of HCs.

Of the 11 included studies, 10 included an analysis of alpha diversity in CPs and HCs, and one did not assess alpha diversity. At the individual study level, the results on the difference in alpha diversity between CPs and HCs were discrepant (**Figure 1**): the Shannon index, which was reported in 10 studies, was found to be significantly lower in CPs than in HCs in six studies and not significantly different in four studies. For further comprehensive analysis of the included studies, we conducted a meta-analysis of the 9 studies that had enough quantitative data. The meta-analysis demonstrated a significant decrease in the Shannon index in the CPs (SMD: -0.53, 95% CI: -0.97 to -0.09) (**Figure 4**). However, the meta-analysis of the Shannon index in the 9 studies using the chi-squared test revealed that there was notable heterogeneity (I^2 ^= 87%, *P <*0.00001). Therefore, sensitivity analysis was performed by omitting each study from the meta-analysis until sufficient homogeneity was achieved (I^2 ^= 0%, *P* = 0.45). After excluding four studies, the meta-analysis of the sensitivity test also demonstrated a significant decrease in CPs (SMD: -1.03, 95% CI: -1.234 to -0.82) (**Figure S1**).

The number of observed operational taxonomic unit (OTUs)/species was significantly lower in CPs ​​than in HCs in all the included studies. The Chao1 index in the CPs ​​was lower than that in the HCs in three out of four studies. The Simpson index in CPs was ​​significantly lower than that in HCs in two out of three studies. The Ace index and Faith’s phylogenetic diversity, which were both reported in only one study, were found to be significantly lower in CPs than in HCs (**Figure 1**).

**Figure 5:**
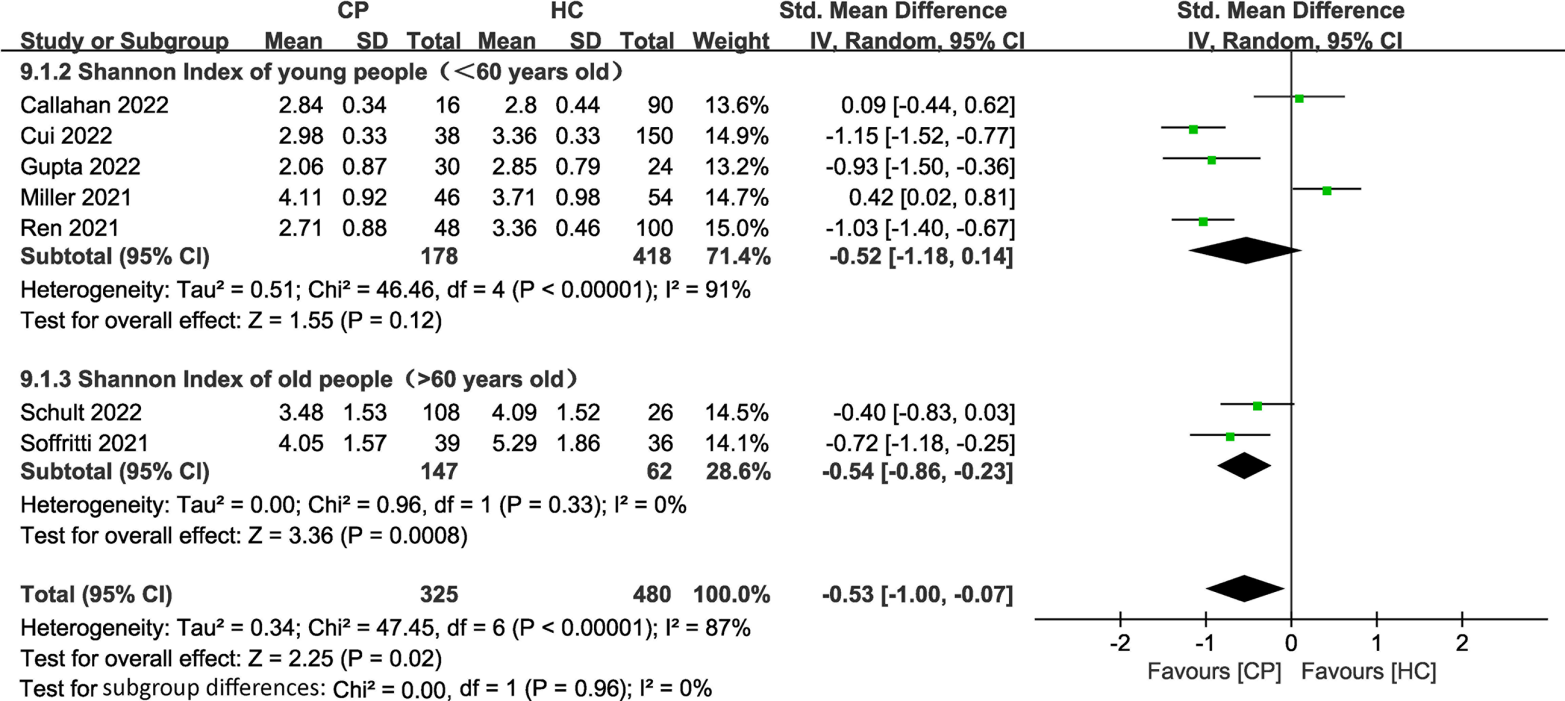
Forest plots of subgroup analysis for Shannon index comparing the oral microbiome of CPs with that of HCs in elderly versus young individuals.

To investigate the impact of age on the alpha diversity in CPs, we divided the included CPs into elderly (> 60 years old) (n=2) (Soffritti et al., 2021; Schult et al., 2022) and young (< 60 years old) (n=5) (Miller et al., 2021; Ren et al., 2021; Callahan et al., 2022; Cui et al., 2022; Gupta et al., 2022) groups according to the World Health Organization’s definition of elderly individuals (Fritz et al., 2017) (two studies without specific age information were excluded). The subgroup meta-analysis (**Figure 5**) demonstrated a significant decrease in the Shannon index in the elderly group (SMD: -0.54, 95% CI: -0.86 to -0.23). The subgroup meta-analysis indicated no significant difference in the Shannon index between young group (SMD: -0.52, 95% CI: -1.18 to 0.14). The chi-squared tests showed that there was adequate homogeneity in the old group (I^2 ^= 0%, *P* =0.33). However, the chi-squared tests showed that there was notable heterogeneity in the young group (I^2 ^= 91%, *P <*0.00001).

To investigate the impact of the use of antibiotics on the alpha diversity in CPs, we divided the included CPs into a group that used antibiotics (at least 1 or more people took antibiotics) (n=4) (Miller et al., 2021; Soffritti et al., 2021; Rafiqul Islam et al., 2022; Schult et al., 2022) and those who did not use antibiotics (none took antibiotics) (n=4) (Ren et al., 2021; Callahan et al., 2022; Gupta et al., 2022; Shi et al., 2022) (one study without specific information on the use of antibiotics was excluded). The subgroup meta-analysis (**Figure S2**) demonstrated no significant differences between the groups (SMD: -0.50, 95% CI: -1.21 to 0.21; SMD: -0.38, 95% CI: -1.09 to 0.33). The chi-squared tests showed that there was notable heterogeneity in the groups that used and did not use antibiotics (I^2 = ^88%, *P <*0.00001)/(I^2 ^= 85%, *P* =0.0002).

#### Beta diversity analysis

Of the 11 included studies, 10 studies investigated beta diversity in CPs and HCs (Iebba et al., 2021; Miller et al., 2021; Wu et al., 2021; Callahan et al., 2022; Cui et al., 2022; Gupta et al., 2022; Rafiqul Islam et al., 2022; Schult et al., 2022; Shi et al., 2022), and one did not assess beta diversity (Soffritti et al., 2021). At the individual study level, the results on the difference in beta diversity between CPs and HCs were basically consistent (**Figure S3**): principal coordinate analysis (PcoA), permutational multivariate analysis of variance (PERMANOVA), analysis of similarities (ANOSIM), and nonmetric multidimensional scaling (NMDS), reported in seven, three, two, and one studies, respectively, showed significant differences between CPs and HCs. Only one PERMANOVA study revealed no significant differences between these two groups.

### Differences in microbial composition at the genus level

**Table 4** shows the differences in the composition of oral microbes between CPs and HCs at the genus level.

As shown in **Figure 2A**, in 11 included studies, a total of 25 bacterial genera had increased abundances in the CPs. Among these studies, six showed that Streptococcus abundance increased (Miller et al., 2021; Soffritti et al., 2021; Cui et al., 2022; Gupta et al., 2022; Rafiqul Islam et al., 2022; Shi et al., 2022), which was the largest increase observed in our study. Campylobacter, Enterococcus, Capnocytophaga, and Klebsiella were found to have significantly higher abundance in CPs than in HCs in two studies. Moreover, twenty genera, including Kingella, Filifactor, Enterobacter, Lactobacillus, Abiotrophias, Atopobium, Parabacteroides, Lachnoclostridium, Megasphaera, Actinomyces, Treponema, Klebsiella, Bacillus, Idiomarina, Acinetobacter, Arenibacter, Chryseobacterium, Bergeyell, Schaalia and Bacteroidete had higher relative abundance in CPs in only one study.

As shown in **Figure 2B**, in 11 included studies, the abundances of a total of 12 bacterial genera decreased in CPs. Among these studies, five showed that Neisseria abundance decreased (Iebba et al., 2021; Wu et al., 2021; Callahan et al., 2022; Gupta et al., 2022; Rafiqul Islam et al., 2022), which was the organism with the largest decrease in abundance observed in our study. Fusobacterium abundance was found to be significantly lower in CPs than in HCs in four studies. Actinobacillus abundance was found to be significantly lower in CPs in three studies. Moreover, tengenera, namely, Corynebacterium, Actinobacillus, Parvimanos, Fusobacterium, Blautia, Faecalibacterium, Ruminococcus, Pseudomonas, Lautropia, and Alloprevotella, had significantly lower abundance in CPs than in HCs in only one study.

In 11 included studies, discrepant results were found regarding 10 bacterial genera, including Veillonella, Porphyromonas, Prevotella, Granulicatella, Aggregatibacter, Rothia, Haemophilus, Gemella, Leptotrichia, and Selenomonas; these bacteria exhibited higher relative abundance in some studies but lower relative abundance in other studies comparing CPs and HCs.

To investigate the impact of age on the microbial composition at the genus level in CPs in the 11 included studies, we evaluated the characteristics of the microbial composition at the genus level in the elderly (> 60 years old) (n=3) (Iebba et al., 2021; Soffritti et al., 2021; Schult et al., 2022) and young (< 60 years old) (n=6) (Miller et al., 2021; Ren et al., 2021; Wu et al., 2021; Callahan et al., 2022; Cui et al., 2022; Gupta et al., 2022) groups (two studies without specific information on age were excluded).

In 3 included studies involving the elderly group, 13 genera, including Enterococcus, Enterobacter, Streptococcus, Veillonella, Prevotella, Lactobacillus, Capnocytophaga, Porphyromonas, Abiotrophia, Aggregatibacter, Atopobium, Parabacteroides and Lachnoclostridium, had higher relative abundance in CPs in only one study, while 9 genera, including Blautia, Faecalibacterium, Ruminococcus, Neisseria, Rothia, Haemophilus, Parvimonas, Fusobacterium and Gemella, had lower relative abundance in CPs in only one study.

In 6 studies that included the young group, a total of 19 bacterial genera had increased abundance in CPs. Among these studies, three showed that Streptococcus and Veillonella abundance increased, which were the largest increases observed in our study. Moreover, seventeen bacterial genera, including Campylobacter, Kingella, Filifactor, Megasphaera, Actinomyces, Treponema, Bacillus, Klebsiella, Idiomarina, Acinetobacter, Arenibacter, Gemella, Chryseobacterium, Capnocytophaga, Bergeyell, Schaalia and Bacteroidete had higher relative abundance in CPs in only one study. In 6 studies that included the young group, a total of 12 bacterial genera had decreased abundance in CPs. Among these studies, four showed that Neisseria abundance decreased, which was the largest decrease observed in this group. Haemophilus, Porphyromonas and Fusobacterium had significantly lower abundance in CPs than in HCs in three studies. Actinobacillus, Aggregatibacter and Rothia had significantly lower abundance in CPs than in HCs in two studies. Moreover, five bacterial genera, including Corynebacterium, Moryella, Alloprevotella, Pseudomonas and Lautropia, had lower relative abundance in CPs in only one study. In 6 included studies, discrepant results were found regarding 4 genera of bacteria, including Granulicatella, Leptotrichia, Selenomonas and Prevotella, which exhibited higher relative abundance in some studies but lower relative abundance in other studies that compared CPs and HCs.

To investigate the impact of the use of antibiotics on the microbial composition at the genus level in CPs in the 11 included studies, we characterized the microbial composition at the genus level in the group that used antibiotics (at least 1 or more people took antibiotics) (n=4) (Miller et al., 2021; Soffritti et al., 2021; Wu et al., 2021; Schult et al., 2022) and the group that did not use antibiotics (none took antibiotics) (n=4) (Iebba et al., 2021; Ren et al., 2021; Gupta et al., 2022; Shi et al., 2022) (one study without specific information on whether antibiotics were used was excluded).

In 4 included studies in which individuals used antibiotics, the abundances of a total of 17 bacterial genera were higher in CPs than in HCs. Among these studies, three showed that Veillonella and Streptococcus abundance increased, were had the largest increases observed in our study. Enterococcus abundance was significantly higher in CPs than in HCs in two studies. Moreover, fourteen bacterial genera, including Campylobacter, Granulicatella, Kingella, Filifactor, Enterobacter, Lactobacillus, Capnocytophaga, Abiotrophia, Atopobium, Parabacteroides, Lachnoclostridium, Actinomyces, Treponema and Klebsiella, had higher relative abundance in CPs in only one study. In 4 studies that included individuals who used antibiotics, a total of 11 bacterial genera exhibited decreased abundance in CPs. Among these studies, two studies showed that Neisseria and Haemophilus abundance decreased, which were the largest decreases observed in this group. Moreover, ten genera, including the bacteria Corynebacterium, Actinobacillus, Moryella, Haemophilus, Fusobacterium, Gemella, Blautia, Faecalibacterium, Ruminococcus and Parvimonas, had lower relative abundance in CPs in only one study. In 4 studies that included individuals who used antibiotics, discrepant results were found regarding 4 bacterial genera, Aggregatibacter, Prevotella, Porphyromonas and Rothia, which exhibited higher relative abundance in some studies but lower relative abundance in other studies that compared CPs and HCs.

In 4 studies that included individuals who did not use antibiotics, a total of 14 bacterial genera had increased abundance in CPs. Among these studies, two studies showed that Gemella and Streptococcus abundance increased, which were the largest increases observed in this group. Moreover, twelve bacterial genera, including Campylobacter, Capnocytophaga, Megasphaera, Klebsiella, Bacillus, Idiomarina, Acinetobacter, Arenibacter, Chryseobacterium, Bergeyell, Schaalia and Bacteroidete, had higher relative abundance in CPs in only one study. In 4 studies that included individuals who did not use antibiotics, a total of 8 bacterial genera had decreased abundance in CPs. Among these studies, three showed that Neisseria and Haemophilus abundance decreased, which were the largest decreases observed in this group. Actinobacillus, Porphyromonas and Fusobacterium had significantly lower abundance in CPs than in HCs in two studies. Moreover, three genera of bacteria, including Pseudomonas, Lautropia and Alloprevotella, had lower relative abundance in CPs in only one study. In 4 studies that included individuals who did not use antibiotics, discrepant results were found regarding 6 genera of bacteria, including Veillonella, Granulicatella, Aggregatibacter, Prevotella, Leptotrichia and Selenomonas, which exhibited higher relative abundance in some studies but lower relative abundance in other studies that compared CPs and HCs.

### Differences in microbial composition at the phylum level

Differences in the relative abundance of oral microbes at the phylum level between CPs and HCs are depicted in **Table 5**. Since four studies did not mention information regarding differences in the relative abundance of bacteria at the phylum level (Iebba et al., 2021; Miller et al., 2021; Wu et al., 2021; Callahan et al., 2022), only 7 studies were included in **Table 5** for analysis (Ren et al., 2021; Soffritti et al., 2021; Cui et al., 2022; Gupta et al., 2022; Rafiqul Islam et al., 2022; Schult et al., 2022; Shi et al., 2022).

As shown in **Figure 2C**, in 7 included studies, regarding the phylum Firmicutes, six studies found higher relative abundance in CPs than HCs (Ren et al., 2021; Soffritti et al., 2021; Gupta et al., 2022; Rafiqul Islam et al., 2022; Schult et al., 2022; Shi et al., 2022). Only one study did not report statistically significant differences between CPs and HCs in the abundances of Firmicutes (Cui et al., 2022). Additionally, another phylum, Fusobacteria, was found to have a significantly higher abundance in CPs in two studies (Ren et al., 2021; Cui et al., 2022).

As shown in **Figure 2D**, in 7 included studies, six phyla, namely, Campilo-bacteriota, Synergistota, Spirochaeota, Patescibacteria, Desulfobacter, and Actinobacteriota, had significantly lower abundance in CPs than in HCs in only one study.

In 7 included studies, discrepant results were found regarding 4 phyla, including Bacteroidetes, Proteobacteria, Actinobacteria and Fusobacteriota, which exhibited higher relative abundance in some studies but lower relative abundance in other studies that compared CPs and HCs.

To investigate the impact of age on the microbial composition at the phylum level in CPs in the 7 included studies, we also evaluated the characteristics of the microbial composition at the phylum level in the elderly (> 60 years old) (n=2) (Soffritti et al., 2021; Schult et al., 2022) and young (< 60 years old) (n=3) (Ren et al., 2021; Callahan et al., 2022; Gupta et al., 2022) groups (two studies without specific information on age were excluded).

In 2 studies that involved the elderly group, a total of 4 bacterial phyla had altered abundances in the CPs. Firmicutes had significantly higher abundance in CPs than in HCs in two studies. Bacteroidetes had significantly higher abundance in CPs than in HCs in one study. Actinobacteria had significantly lower abundance in CPs than in HCs in one study. Proteobacteria showed discrepant abundance results in the 2 included studies.

In 3 studies that involved the young group, a total of 10 bacterial phyla exhibited differences in the CPs. Firmicutes had significantly higher abundance in CPs than in HCs in two studies. Bacteroidetes and Proteobacteria had significantly lower abundance in CPs than in HCs in two studies. Six bacterial phyla, including Actinobacteriota, Campilo-bacteriota, Synergistota, Spirochaeota, Patescibacteria and Desulfobacter, had lower relative abundance in CPs in only one study. Fusobacteria showed discrepant abundance results in 3 included studies.

To investigate the impact of the use of antibiotics on the microbial composition at the phylum level in CPs in the 7 included studies, we also evaluated the characteristics of the microbial composition at the phylum level in the group that used antibiotics (at least 1 or more people took antibiotics) (n=3) (Soffritti et al., 2021; Rafiqul Islam et al., 2022; Schult et al., 2022) and those who did not use antibiotics (none took antibiotics) (n=3) (Ren et al., 2021; Gupta et al., 2022; Shi et al., 2022) (one study without specific information of whether antibiotics were used was excluded).

In 3 studies that included individuals who used antibiotics, a total of 3 bacterial phyla had higher abundance in CPs than in HCs. Among these studies, three showed that Firmicutes abundance increased, which was the largest increase observed in our study. Bacteroidetes and Firmicutes had significantly higher abundance in CPs than in HCs in one study. In 3 studies that included individuals who used antibiotics, Actinobacteria had significantly lower abundance in CPs than in HCs in one study. In 3 studies that included individuals who used antibiotics, discrepant results were found regarding Proteobacteria; they exhibited a higher relative abundance in some studies but a lower relative abundance in other studies that compared CPs and HCs.

In 3 studies that included individuals who did not use antibiotics, a total of 3 bacterial phyla had higher abundance in CPs than in HCs. Among the studies, three showed that Firmicutes abundance increased, which was the largest increase observed in our study. Fusobacteria had significantly higher abundance in CPs than in HCs in two studies. Actinobacteria had significantly higher abundance in CPs than in HCs in one study. In 3 studies that included individuals who did not use antibiotics, Proteobacteria had significantly lower abundance in CPs than in HCs in three studies. Moreover, five bacterial phyla, including Bacteroidetes, Fusobacteriota, Campilo-bacteriota, Synergistota and Spirochaeota, had lower relative abundance in CPs in only one study. In 3 studies that included individuals who did not use antibiotics, discrepant results were found regarding Patescibacteria; they exhibited a higher relative abundance in some studies but exhibited a lower relative abundance in other studies that compared CPs and HCs.

### Bias assessment

Since seven studies did not address potential confounding factors, such as age, sex, antibiotic intake, and severity of COVID-19 (as shown in **Table 2**, **Table S3**) (Miller et al., 2021; Soffritti et al., 2021; Wu et al., 2021; Cui et al., 2022; Rafiqul Islam et al., 2022; Schult et al., 2022; Shi et al., 2022), a serious risk of bias was mainly found in the domain of confounding. Of the 11 studies, 1 was considered to have moderate overall bias, and 10 were considered to have serious overall bias. Overall, the results of the meta-analyses indicated a serious risk of bias (**Table S4**).

### DPP4 and ACE2 expression levels in oral tissues

**Figure 6:**
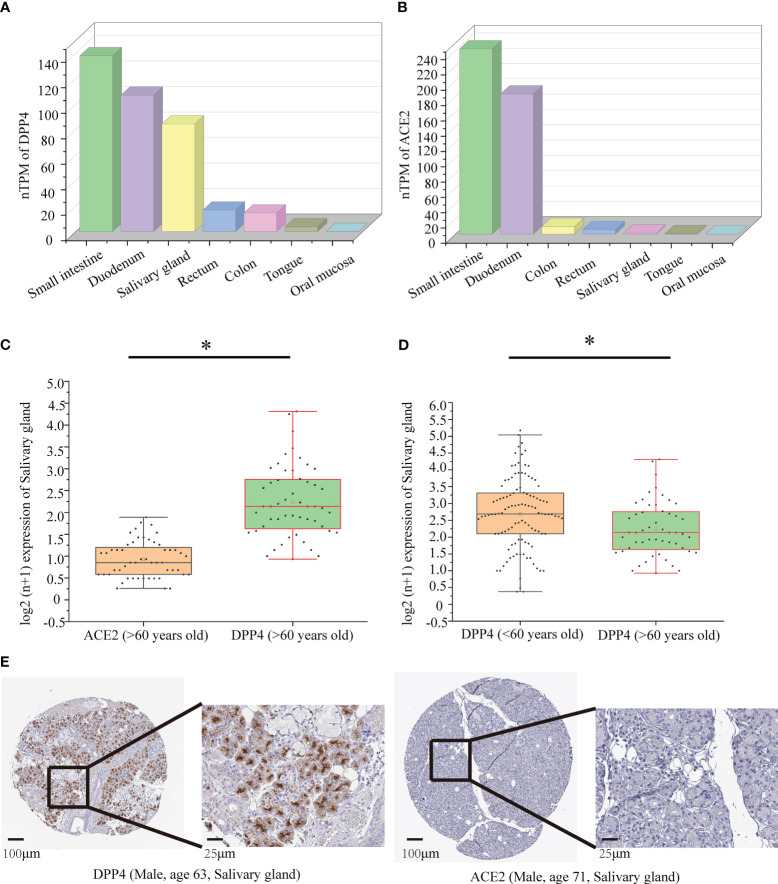
Protein and mRNA expression of DPP4 and ACE2 based on the GTEx database. **(A)** The mRNA level of DPP4 in gut and oral tissues from GTEx samples; **(B)** The mRNA level of ACE2 in gut and oral tissues from GTEx samples; **(C)** The mRNA level of DPP4 and ACE2 in the salivary gland in the elderly (> 60 years old) group; **(D)** The mRNA level of DPP4 in the salivary gland in the young (< 60 years old) versus elderly (> 60 years old) group. **(E)** Immunohistochemical images of DPP4 and ACE2 expression in the salivary glands in the elderly (> 60 years old) group. TPM, transcripts per million. The Log2 (n+1) scale was used for visualization. Significant differences between the groups are indicated by an asterisk (*) with p < 0.05 (independent t-tests).

By using data collected from the HPA database, we found that the highest expression of ACE2 and DPP4 in intestinal tissues in healthy people was in the small intestine and duodenum. However, most oral tissues do not express ACE2, and DPP4 only has meaningful expression levels in the oral salivary gland, not in other oral tissues, such as the tongue and oral mucosa (**Figure 6A, B**). Therefore, we chose to investigate the oral salivary gland in the follow-up studies, and the DPP4 and ACE2 mRNA levels in a total of 324 oral salivary gland tissues were analysed. Since the decrease in oral microbiome alpha diversity in the elderly (> 60 years old) group was the most obvious in this study, we divided the samples into young (< 60 years old) and elderly (> 60 years old) groups; the mRNA expression level of DPP4 was much higher than that of ACE2 in the healthy human oral salivary gland in the elderly (p < 0.05) (**Figure 6C**). We also found that the mRNA expression level of DPP4 was significantly lower in the elderly group than in the young group (p < 0.05) (**Figure 6D**). To evaluate the protein expression of DPP4 and ACE2 in the oral salivary gland tissues in elderly individuals, we downloaded immunohistochemistry images from the HPA database. As shown in **Figure 6E**, the expression level of the DPP4 protein was significantly higher in the oral salivary gland tissues in elderly individuals, while no positive staining for ACE2 protein expression was observed.

### Analysis of the correlation between DPP4 expression and the expression of viral genes

**Figure 7:**
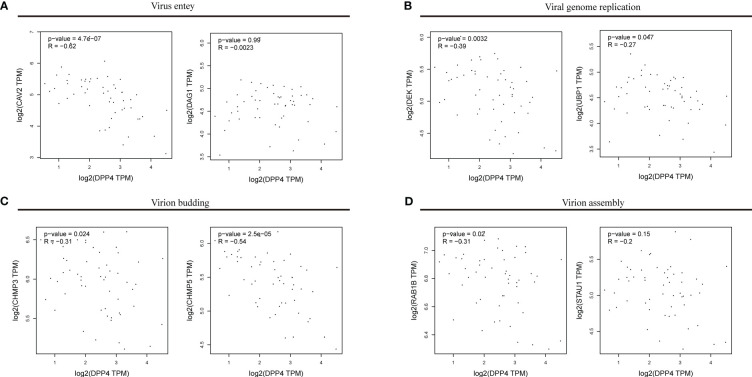
Correlations between DPP4 levels and the expression of viral genes. The x-axis shows DPP4 expression, and the y-axis shows the expression of genes involved in **(A)** viral entry, **(B)** viral genome replication, **(C)** virion budding, **(D)** and virion assembly. Each dot represents a single sample from the normal human left ventricle. TPM, transcripts per million. The log2 scale was used for visualization, and R represents the correlation coefficient of Spearman’s analysis. DPP4, dipeptidyl peptidase 4.

To explore the correlation between DPP4 expression and viral infection, we selected a variety of gene markers involved throughout viral processes. These gene markers were input into GEPIA, and pairwise gene correlation was performed using DPP4 levels. Spearman correlation analysis showed that DPP4 expression was negatively correlated with the expression of viral genes, such as those involved in entering host cells, virus genome replication, virus assembly, and budding (**Figure 7**). Notably, we found that the viral genes involved in entering the host cell, such as dystroglycan 1 (DAG1), had negative correlations with DPP4 levels (**Figure 7A**). Furthermore, DPP4 mRNA levels were more negatively correlated with the levels of genes involved in viral genome replication, virion assembly and budding-related genes containing upstream binding protein 1 (UBP1), ubiquitous phosphorylated nuclear protein (DEK), charged multivesicular body protein 3 (CHMP3), charged multivesicular body protein 5 (CHMP5), and Ras-related protein Rab-1B (RAB1B) (**Figures 7B–D**).

### DPP4 protein network analysis and gene enrichment analysis

**Figure 8:**
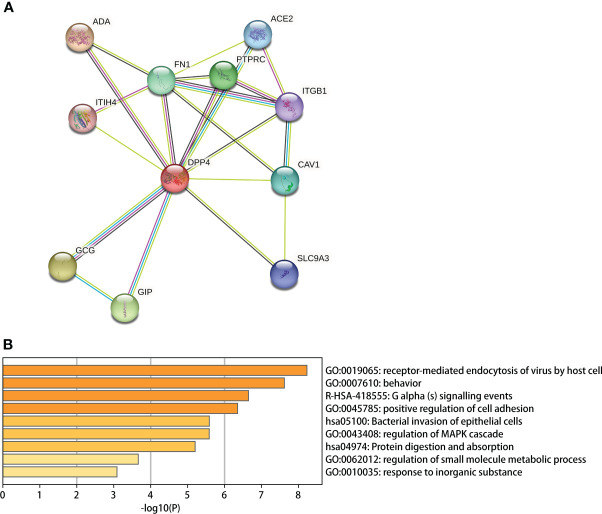
DPP4 protein network analysis and gene enrichment analysis. **(A)** PPI network establishment based on the 10 proteins with the highest score in combination with DPP4 in STRING; **(B)** A Gene enrichment analysis for the biological processes in which DPP4 and its 10 predicted partners are involved.

We used the STRING site to explore the interaction between DPP4 levels and the top 10 predicted high combined score proteins for further analysis (**Table 6**). The PPI network structure generated using 10 related proteins is shown in **Figure 8A**. To annotate the functions of the 10 proteins, we performed gene enrichment analysis using Metascape. The top 9 significant gene enrichment terms of biological processes are illustrated in **Figure 8B**. The results of gene enrichment analysis showed that DPP4-associated proteins were mainly enriched in the regulation of receptor-mediated endocytosis of viruses by host cells, which was basically consistent with the original function of DPP4. In addition, DPP4-related proteins were significantly enriched for some biological processes, such as bacterial invasion of epithelial cells and regulation of small molecule metabolic processes.

**Table 6:** Top 10 predicted partner proteins of DPP4 in STRING.

Gene symbol	Full name	Combined score
ADA	Adenosine deaminase	0.997
GCG	Glucagon	0.993
GIP	Gastric inhibitory polypeptide	0.992
PTPRC	Receptor-type tyrosine-protein phosphatase C	0.991
FN1	Fibronectin 1	0.990
CAV1	Caveolin-1	0.988
ACE2	Angiotensin-converting enzyme 2	0.980
SLC9A3	Sodium/hydrogen exchanger 3	0.979
ITGB1	Integrin beta-1	0.975
ITIH4	Inter-alpha-trypsin inhibitor heavy chain family member 4	0.972

Top 10 predicted partner proteins of DPP4 in STRING (Homo sapiens).

## Discussion

In general, the results show wide variation in oral microbial composition in COVID-19 patients. Changes in alpha and beta diversity parameters (**Figure 1**; **Figure S3**) showed that the oral microbiome in SARS-CoV-2-infected individuals in most studies was dramatically different from that in noninfected individuals. Specifically, the meta-analysis demonstrated a significant decrease in the Shannon index in the CPs (SMD: -0.53, 95% CI: -0.97 to -0.09) (**Figure 4**). However, the chi-squared tests showed that there was notable heterogeneity (I^2 ^= 87%, *P <*0.00001). Therefore, sensitivity analysis was performed by omitting each study from the meta-analysis until sufficient homogeneity was achieved (I^2 ^= 0%, *P* = 0.45) (**Figure S1**). After the exclusion of four studies, the meta-analysis of the sensitivity test result also demonstrated a significant decrease in CPs (SMD: -1.03, 95% CI: -1.234 to -0.82) (**Figure S1**). Since a decline in the Shannon index indicates a decline in alpha diversity (Lüll et al., 2021), we revealed that there is lower alpha diversity in CPs by screening our meta-analysis results.

The lower alpha diversity and changing beta diversity have been suggested as key features of microbiome dysbiosis, which is usually related to an increase in the levels of opportunistic pathogens and immune dysregulation in several diseases (Song et al., 2020; Varricchi et al., 2021; Shi et al., 2022). Much evidence suggests that dysbiosis of the oral microbiome can cause the growth of opportunistic pathogens that can produce bacterial toxins such as dentilisin (from mammalian oral Treponema), Fusobacterial toxins, and Pasteurella multocida toxin and promote chronic inflammation and immune-subversion by modulating cell proliferation, replication and death (Joossens et al., 2011; Marchesi et al., 2011; Hajishengallis and Lamont, 2014). Dysbiosis of the oral microbiome was also found to be linked with different clinical manifestations of COVID-19, including loss of taste, breathing difficulty, and sore throat (Rafiqul Islam et al., 2022), which have recently been shown to not be caused by direct viral damage but by chronic inflammation and immune-subversion (Gupta and Gupta, 2021; Ho et al., 2022). Therefore, we believe that improving dysbiosis of the oral microbiome is crucial to improve the chronic inflammation and immune-subversion that occurs in patients with COVID-19.

The subgroup meta-analysis of our results demonstrated a significant decrease in the Shannon index in the elderly group (SMD: -0.54, 95% CI: -0.86 to -0.23) with adequate homogeneity (I^2 ^= 0%, *P* =0.33). The subgroup meta-analysis showed no significant difference in the young group (SMD: -0.52, 95% CI: -1.18 to 0.14), with notable heterogeneity (I^2 ^= 91%, *P <*0.00001) in the Shannon index (**Figure 5**). These results emphasize that the elderly are the main population that exhibit lower alpha diversity and more serious dysbiosis of the oral microbiome among CPs. A previous meta-analysis confirmed that increased age (≥65 years old) was associated with high mortality in COVID-19 (Parohan et al., 2020), and the study suggested that the main reason for high mortality in elderly individuals is that age-dependent defects in B-cell and T-cell function could lead to prolonged proinflammatory responses and deficiency in the control of viral replication (Opal et al., 2005). Therefore, we believe that the side effects mentioned above, such as an increase in the levels of opportunistic pathogens and immune dysregulation caused by dysbiosis of the oral microbiome, may accelerate this process and further lead to coinfection and eventual death in elderly COVID-19 patients. This concept is also consistent with previous studies that found that oral health intervention during pneumonia reduced mortality in patients and further emphasizes that it is essential to regulate oral microbial homeostasis in elderly COVID-19 patients (Mori et al., 2006; Manger et al., 2017).

The subgroup meta-analysis demonstrated no significant difference in alpha diversity between groups treated with or without antibiotics (SMD: -0.50, 95% CI: -1.21 to 0.21; SMD: -0.38, 95% CI: -1.09 to 0.33) (**Figure S2**). Studies indicate that antibiotics are prescribed for COVID-19 patients mainly to prevent coinfection with opportunistic pathogens (Langford et al., 2020; Lansbury et al., 2020; Rawson et al., 2020). As the results of this study show that the use of antibiotics does not cause serious dysfunction of the oral microbiota, we believe that preventive use is still necessary, especially to prevent the high mortality caused by COVID-19 combined with early-onset bacterial coinfection (Elabbadi et al., 2021).

According to our study, Streptococcus is the genus with the highest abundance in CPs regardless of age or whether antibiotics were used. Some species of Streptococcus are opportunistic pathogens responsible for several diseases that act by stimulating or inhibiting immune defences mounted against them (Nobbs et al., 2009). Specifically, Streptococcus that colonize mucosal tissues in the oral cavity can not only change the microbial composition of the respiratory system but also promote a series of cytokine responses, such as those mediated by IL-6 and IL-8, and affect the immune homeostasis of the lungs (Bao et al., 2020). Under certain conditions, Streptococcus gordonii can attack host fibronectin, and subsequent cytokine production can induce inflammatory responses (Erb-Downward et al., 2011; Lu et al., 2017). In studies of other diseases, such as rheumatoid arthritis, Streptococcus was also determined to be a major contributor to dysbiosis of the oral microbiota, and their cell walls can influence innate immunity and aggravate disease by inducing the production of inflammatory factors (Moentadj et al., 2021). Therefore, it is necessary to prevent infections by Streptococcus and other potential opportunistic pathogens, especially in elderly individuals with low immunity (Gaillat, 2003). However, the main strategies used to prevent infection still have limitations. For example, vaccination against Streptococcus is less immunogenic and efficient in the elderly because of age-related changes in the immune system (Weinberger and Grubeck-Loebenstein, 2012), and Streptococcus easily develops antimicrobial resistance to many first-line antibiotics, such as penicillins, macrolides and tetracyclines (Haenni et al., 2018). This phenomenon implies that more effective potential interventions are needed to reduce the increase in the levels of Streptococcus and other potential opportunistic pathogens involved in oral microbiota dysfunction, such as oral health intervention during COVID-19.

Neisseria is the genus with the lowest abundance in CPs regardless of age or whether antibiotics are used. The Neisseria genus is reported to be the fourth most abundant bacterial genus in the oral microbiota of adults and can maintain the stability of the human immune system. For instance, nonpathogenic Neisseria species are thought to have a physiological role in preventing the colonization of oral and nasal sites by potential pathogens and are also important in developing the T-cell-independent polyclonal IgM response and maintaining immune ignorance in the acquired immune response (Dorey et al., 2019). A study also reported that dysbiosis of the oral microbiota after infection with SARS-COV-2 was attributed to a decrease in Neisseria abundance because this bacterium suppresses important metabolic pathways, such as the host tricarboxylic acid cycle (Wu et al., 2021). Therefore, based on our research results, maintaining the normal level of Neisseria in COVID-19 patients may be crucial to maintain the stability of the oral microbiota, which can also further improve immune dysregulation and prevent bacterial coinfection in COVID-19 patients.

Moreover, determining the reasons that dysbiosis of the oral microbiome is more likely to occur in elderly individuals remains a serious issue, but until now, there had been no research to clarify the mechanism of this phenomenon. DPP4 and ACE2 are the main host cell receptors of 2019-nCoV and can be expressed in many types of cells, such as enterocytes and cardiomyocytes (Hikmet et al., 2020; Postlethwait et al., 2021). The expression of DPP4 and ACE2 in enterocytes in the intestine is closely related to gut microbiome dysbiosis in COVID-19, but the specific mechanism is still unclear (Vuille-dit-Bille et al., 2015; Olivares et al., 2018; Posadas-Sánchez et al., 2021; Yu et al., 2021). Therefore, we decided to explore whether DPP4 and ACE2 have similar effects in regulating the oral microbiome.

We first determined that the highest expression of DPP4 in the oral cavity was in oral salivary gland tissue by using the GTEx database on the HPA website (**Figures 6A, B**). Since the decrease in oral microbiome alpha diversity in the elderly (> 60 years old) was the most obvious in this study, we further divided 324 oral salivary gland tissues into young (< 60 years old) and elderly (> 60 years old) groups and found that the mRNA and protein expression levels of DPP4 were extremely high, while ACE2 expression was almost undetectable in the healthy human oral salivary gland of elderly individuals (p < 0.05) (**Figures 6C, E**). We further found that the mRNA expression level of DPP4 was significantly lower in the elderly group than in the young group (p < 0.05) (**Figure 6D**). Recent research has shown that ageing is associated with changes in the numbers of receptors for 2019-nCoV, such as ACE2, which is related to an increased risk of death from COVID-19 (Farshbafnadi et al., 2021). Our results demonstrated that ageing is also associated with a decline in DPP4 expression in oral salivary glands, which may lead to susceptibility of elderly individuals to viruses because DPP4 expression was negatively correlated with many viral genes, including those involved in host cell entry (CAV2), viral genome replication (DEK, UBP1), virion assembly (CHMP3, CHMP5), and budding (RAB1B) (**Figure 7**) (Zhao et al., 2020). Furthermore, we found that the decreased expression of DPP4 in the oral salivary glands of elderly individuals may be closely related to viral infection and side effects of dysbiosis of the oral microbiome by conducting gene enrichment analysis (**Figure 8B**) because DPP4-associated proteins were mainly enriched in biological processes such as regulation of receptor-mediated endocytosis of viruses by host cells and bacterial invasion of epithelial cells. This finding is also consistent with a previous study that found that a low level of DPP4 in serum is strongly related to a high risk of death from COVID-19 (Posadas-Sánchez et al., 2021). Therefore, we hypothesized that the lower expression of DPP4 in the oral salivary gland tissues in elderly people than in young people is closely related to the aggravation of COVID-19 and dysfunction of the oral microbiota (as ACE2 can play a similar role in enterocytes of the intestine of COVID-19 patients). However, in the future, this idea needs further discussion and proof.

Overall, the immunopathogenesis of SARS-CoV-2 and associated mechanisms of coinfection are still not clearly understood (Patel and Sampson, 2020; Bortolotti et al., 2021). However, dysbiosis of the oral microbiota may play a major role in not only affecting innate immunity but also increasing the risk of bacterial coinfection during and death from COVID-19 (Yu et al., 2019; Bao et al., 2020). Especially in elderly individuals, the decrease in DPP4 expression in oral salivary gland tissues may also participate in the whole process. Therefore, we believe that it is of great importance to develop a clinical strategy to improve dysbiosis of the oral microbiota, which may reduce immune dysregulation and bacterial coinfection and further reduce the mortality of COVID-19.

Finally, a few studies have documented the influence of the severity of COVID-19 on dysbiosis of the oral microbiota. Therefore, it is necessary to conduct more studies that are focused on the relationship between the severity of COVID-19 and dysbiosis of the oral microbiota in COVID-19 patients. Moreover, there are still many confounding factors that will affect conclusions regarding the changes in the composition of the oral microbiome in CPs, such as the use of antibiotics, periodontal disease, and differences in the maintenance of oral hygiene during hospitalization. This research requires us to standardize the clinical parameters of included patients and eliminate interfering factors as much as possible to better study the effect of the oral microbiota on COVID-19.

## Conclusion

This study showed that the oral microbial composition of COVID-19 patients was significantly altered compared to normal individuals, especially among the elderly. In addition, DPP4 in the oral of elderly was significantly down regulated, which may be related to viral infection and oral microbiome dysbiosis. It suggests that clinical strategies to improve oral microbiota dysbiosis may play a key role in reducing immune dysregulation, bacterial co-infection and further reducing mortality in COVID-19 patients.

## Data availability statement

The original contributions presented in the study are included in the article/**Supplementary Material**. Further inquiries can be directed to the corresponding authors.

## Author contributions

LT was responsible for research conceptualization and task management. M-MZ, QL, YC, Y-QZ were responsible for research selection and data obtain. Y-HZ, YF, QY, JH, Z-YO-Y, MDA and JZ conducted the statistical analyses. YG and Y-ZF supervised the project. All authors contributed to the article and approved the submitted version.
