# Supplementary figures and images for: Potential interaction between the oral microbiota and COVID-19: a meta-analysis and bioinformatics prediction
Source: Front Cell Infect Microbiol. 2023 Jun 7;13:1193340. doi: 10.3389/fcimb.2023.1193340 (PMC10282655; doi:10.3389/fcimb.2023.1193340)

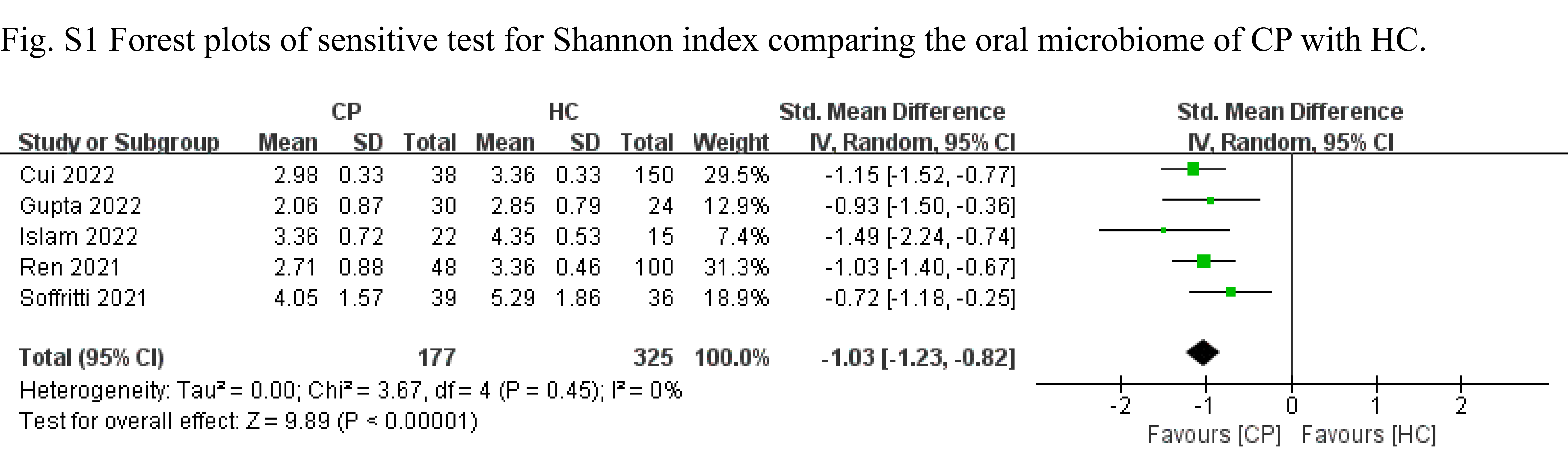

Supplement: Supplementary file 1 [file Image_1.tif]

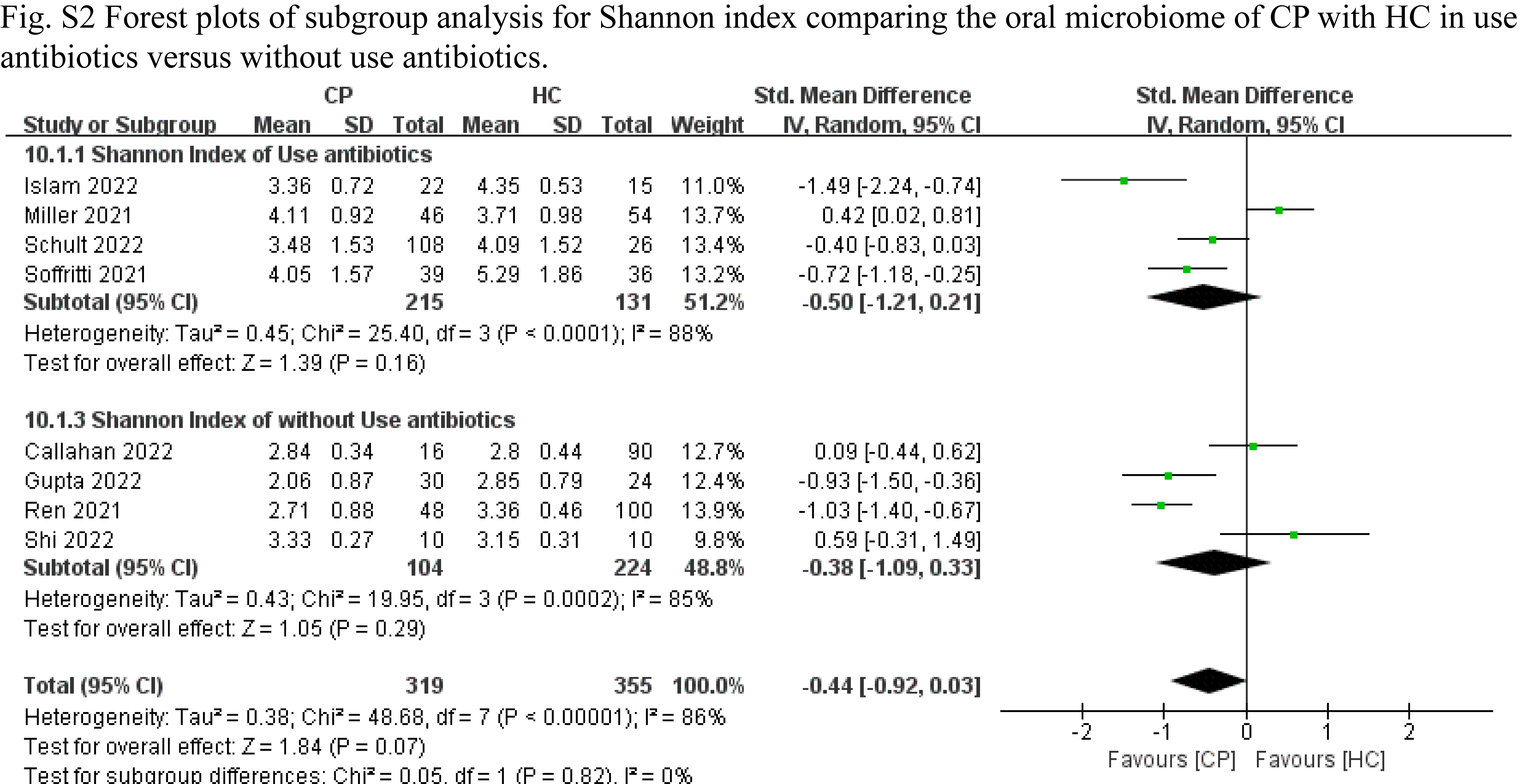

Supplement: Supplementary file 2 [file Image_2.tif]

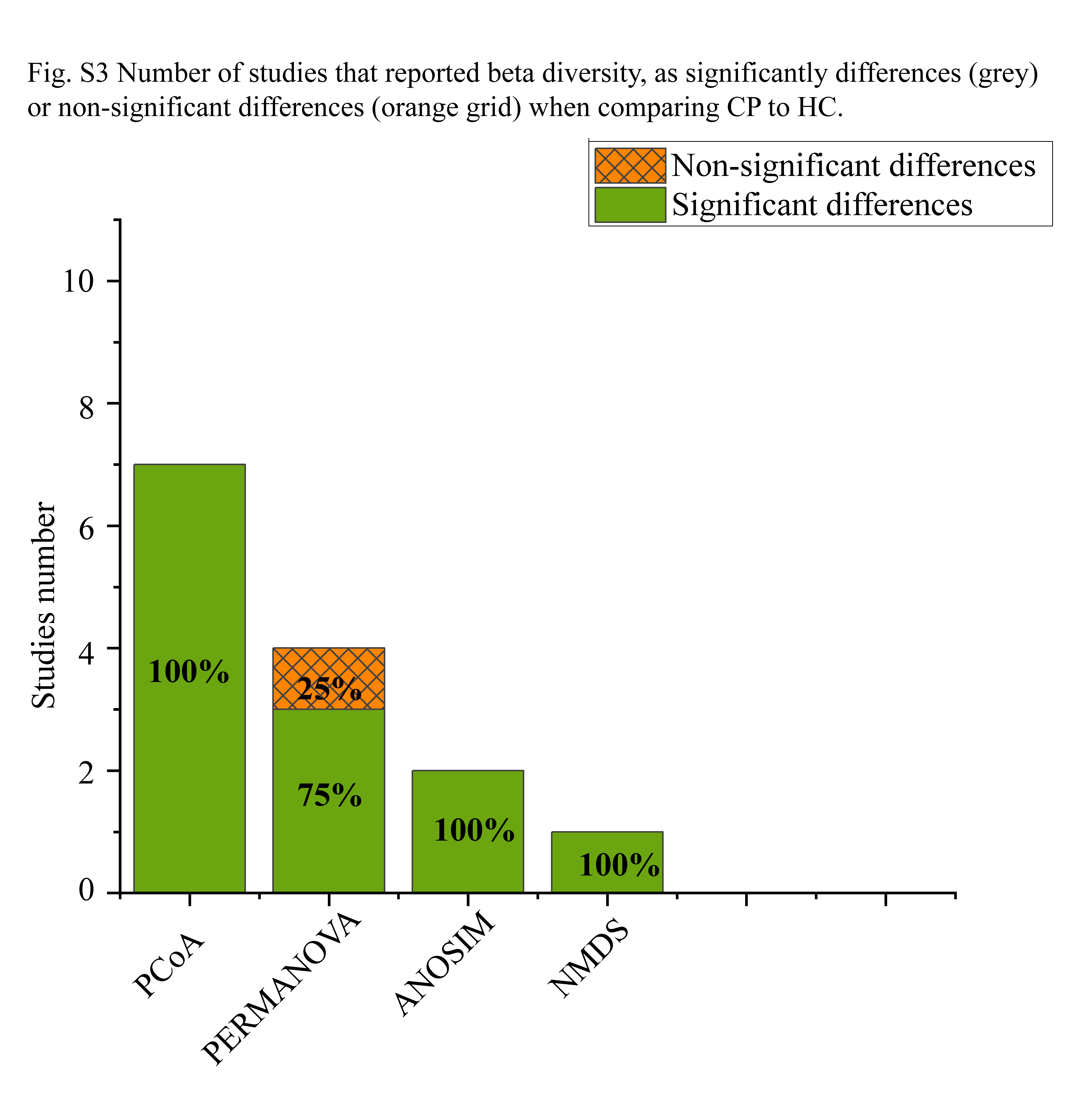

Supplement: Supplementary file 3 [file Image_3.tif]
