# Supplementary material for: Potential interaction between the oral microbiota and COVID-19: a meta-analysis and bioinformatics prediction
Source: Front Cell Infect Microbiol. 2023 Jun 7;13:1193340. doi: 10.3389/fcimb.2023.1193340 (PMC10282655; doi:10.3389/fcimb.2023.1193340)
Supplement: Supplementary file 4 [file Table_1.docx]

**Table S1** Embase search strategy

| PubMed | | Search Strategy (October, 2022) | | | | | Items |
| --- | --- | --- | --- | --- | --- | --- | --- |
| #1 | 'severe acute respiratory syndrome coronavirus 2'/exp OR 'coronavirus disease 2019'/exp OR 'coronavirus disease 2019':ab,ti OR 'ncov':ab,ti OR 'novel coronavirus':ab,ti OR 'coronavirus':ab,ti OR 'wuhan coronavirus':ab,ti OR 'sars2':ab,ti OR 'sars cov 2':ab,ti OR 'severe acute respiratory syndrome coronavirus 2':ab,ti OR 'covid 19':ab,ti | | | | | |  |
|  |  |  |  |  |  |  | 356252 |
|  |  |  |  |  |  |  |  |
|  |  |  |  |  |  |  |  |
| #2 | 'mouth flora'/exp OR 'oral microbiome':ab,ti OR 'oral flora':ab,ti OR 'oral bacteria':ab,ti OR 'oral microbiota':ab,ti | | | | | |  |
|  |  |  |  |  |  |  | 72040 |
|  |  |  |  |  |  |  |  |
| #3 |  |  |  |  |  |  |  |
|  | #1 AND #2 | | | | | | 99 |
|  |  |  |  |  |  |  |  |
