# Supplementary material for: Potential interaction between the oral microbiota and COVID-19: a meta-analysis and bioinformatics prediction
Source: Front Cell Infect Microbiol. 2023 Jun 7;13:1193340. doi: 10.3389/fcimb.2023.1193340 (PMC10282655; doi:10.3389/fcimb.2023.1193340)
Supplement: Supplementary file 5 [file Table_2.docx]

**Table S2** Cochrane Library search strategy

| Cochrane Library | | Search Strategy (October, 2022) | | | | | Items |
| --- | --- | --- | --- | --- | --- | --- | --- |
| #1 | Mesh descriptor: [COVID-19] explode all trees OR Mesh descriptor: [SARS-CoV-2] explode all trees OR (COVID-19):ti,ab,kw OR (severe acute respiratory syndrome coronavirus 2):ti,ab,kw OR (SARS-Cov-2):ti,ab,kw OR (SARS2):ti,ab,kw OR (wuhan coronavirus):ti,ab,kw OR (coronavirus):ti,ab,kw OR (novel coronavirus):ti,ab,kw OR (nCoV):ti,ab,kw OR (coronavirus disease 2019):ti,ab,kw | | | | | |  |
|  |  |  |  |  |  |  | 12950 |
|  |  |  |  |  |  |  |  |
|  |  |  |  |  |  |  |  |
| #2 | Mesh descriptor: [Microbiota] explode all trees OR (oral microbiome):ti,ab,kw OR (oral flora):ti,ab,kw OR (oral bacteria):ti,ab,kw OR (oral microbiota):ti,ab,kw | | | | | |  |
|  |  |  |  |  |  |  | 6089 |
|  |  |  |  |  |  |  |  |
| #3 |  |  |  |  |  |  |  |
|  | #1 AND #2 | | | | | | 35 |
|  |  |  |  |  |  |  |  |
