# Supplementary material for: Potential interaction between the oral microbiota and COVID-19: a meta-analysis and bioinformatics prediction
Source: Front Cell Infect Microbiol. 2023 Jun 7;13:1193340. doi: 10.3389/fcimb.2023.1193340 (PMC10282655; doi:10.3389/fcimb.2023.1193340)
Supplement: Supplementary file 6 [file Table_3.docx]

**Table S3** Characteristics of Severity of COVID-19 and Antibiotic Use of included studies.

| Studies | Information about control group | Common Comorbidity | Severity of COVID-19 | Antibiotic Use ratio in COVID-19 patients | Criteria for judging the severity of COVID-19 |
| --- | --- | --- | --- | --- | --- |
| Wu et al., 2021 | Healthy controls | Not mentioned | 90 non-severe and 50 severe COVID-19 patients | 67.9% | The instruction of the New Coronavirus Pneumonia Prevention and Control Program (7th edition) published by the National Health Commission of China） |
| Soffritti et al., 2021 | Control group affected by non-respiratory diseases | Not mentioned | 11 asymptomatic, 7 mild symptoms, and 21 symptomatic COVID-19 patients | 100% (hydroxychloroquine and azithromycin on hospitalization) | COVID-19 patients were stratified into  Four categories based on symptoms: asymptomatic (1, no  symptoms), paucisymptomatic (2, aspecific flu-like symptoms),  symptomatic (3, including specific respiratory symptoms),  severely symptomatic (4, needing ventilation). |
| Shi et al., 2022 | Healthy controls | Not mentioned | 10 mild COVID-19 patients | 0% (without using antibiotics > 2 months) | The severity of COVID-19 infection was categorized as mild according to Ct. value |

**Table S3** Continued.

| Studies | Information about healthy control group | Common Comorbidity | Severity of COVID-19 | Antibiotic Use ratio in COVID-19 patients | Criteria for judging the severity of COVID-19 |
| --- | --- | --- | --- | --- | --- |
| Schult et al., 2022 | 26 age- and gender-matched asymptomatic controls (AC) | Hypertension（39.8%） | 68 mild, 22 severe, 17death COVID-19 patients | 50% | Patients with COVID-19 were  classified based on the WHO  ordinal scale for clinical improvement for hospitalized patients with COVID-19 |
| Ren et al., 2021 | Healthy controls | Not mentioned | Not mentioned | 0% (without using antibiotics > 8 weeks) | Not mentioned |
| Miller et al., 2021 | Healthy controls | Hypertension（54.7%） | Not mentioned | 47.2% | Not mentioned |

**Table S3** Continued.

| Studies | Information about control group | Common Comorbidity | Severity of COVID-19 | Antibiotic Use ratio in COVID-19 patients | Criteria for judging the severity of COVID-19 |
| --- | --- | --- | --- | --- | --- |
| Islam et al., 2022 | Healthy controls | Not mentioned | Not mentioned | 50% (azithromycin, metronidazole, amoxicillin, and ciprofloxacin) | Not mentioned |
| Iebba et al., 2021 | Age-matched healthy controls | Not mentioned | Not mentioned | 0% (without using antibiotics > 3 months) | Not mentioned |
| Gupta et al., 2022 | Healthy controls | Diabetes, chronic renal disease, and hypertension (40%) | 10 symptomatic and 20 asymptomatic patients | 0% | Not mentioned |

**Table S3** Continued.

| Studies | Information about control group | Common Comorbidity | Severity of COVID-19 | Antibiotic Use ratio in COVID-19 patients | Criteria for judging the severity of COVID-19 |
| --- | --- | --- | --- | --- | --- |
| Cui et al., 2021 | Gender and age-matched healthy controls | Not mentioned | Recover patients | Not mentioned | Confrmed patients recover at  discharge |
| Callahan et al., 2022 | Healthy controls | Not mentioned | Not mentioned | 0% (All patients did not take antibiotics > 1 month) | Not mentioned |
