# Supplementary material for: Potential interaction between the oral microbiota and COVID-19: a meta-analysis and bioinformatics prediction
Source: Front Cell Infect Microbiol. 2023 Jun 7;13:1193340. doi: 10.3389/fcimb.2023.1193340 (PMC10282655; doi:10.3389/fcimb.2023.1193340)
Supplement: Supplementary file 7 [file Table_4.docx]

**Table S4** Risk of bias for each study

| Risk of Bias | **Selection of participants** | **Confounding variables** | **Measurement of exposure** | **Blinding of outcome assessment** | **Incomplete outcome data** | **Selective outcome reporting** |
| --- | --- | --- | --- | --- | --- | --- |
| Wu et al., 2021 | Low | Unclear | Low | Low | Low | Unclear |
| Soffritti et al., 2021 | Low | Unclear | Low | Low | Low | Unclear |
| Shi et al., 2022 | Unclear | Unclear | Low | Low | Low | Unclear |
| Schult et al., 2022 | Low | Unclear | Low | Low | Low | Unclear |
| Ren et al., 2021 | Low | Unclear | Low | Low | Low | Unclear |
| Miller et al., 2021 | Low | Unclear | Low | Low | Low | Unclear |
| Islam et al., 2022 | Low | Unclear | Low | Low | Low | Unclear |
| Iebba et al., 2022 | Unclear | Unclear | Low | Low | Low | Unclear |
| Gupta et al., 2022 | Unclear | Unclear | Low | Low | Low | Unclear |
| Cui et al., 2021 | Unclear | Unclear | Unclear | Low | Unclear | Unclear |
| Callahan et al., 2022 | Unclear | Unclear | Unclear | Low | Unclear | Unclear |
